# Use of Multiple Low Cost Carbon Dioxide Sensors to Measure Exhaled Breath Distribution with Face Mask Type and Wearing Behaviour

**DOI:** 10.3390/s21186204

**Published:** 2021-09-16

**Authors:** Naveed Salman, Muhammad Waqas Khan, Michael Lim, Amir Khan, Andrew H. Kemp, Catherine J. Noakes

**Affiliations:** 1 School of Civil Engineering, University of Leeds, Leeds LS2 9JT, UK; a.khan@leeds.ac.uk (A.K.); c.j.noakes@leeds.ac.uk (C.J.N.); 2 Engineering & Environment, Northumbria University, Newcastle NE1 8ST, UK; m.w.khan@northumbria.ac.uk (M.W.K.); michael.lim@northumbria.ac.uk (M.L.); 3 School of Electronic and Electrical Engineering, University of Leeds, Leeds LS2 9JT, UK; a.h.kemp@leeds.ac.uk

**Keywords:** face mask, CO_2_ sensors, COVID-19, data interpolation

## Abstract

The use of cloth face coverings and face masks has become widespread in light of the COVID-19 pandemic. This paper presents a method of using low cost wirelessly connected carbon dioxide (CO_2_) sensors to measure the effects of properly and improperly worn face masks on the concentration distribution of exhaled breath around the face. Four types of face masks are used in two indoor environment scenarios. CO_2_ as a proxy for exhaled breath is being measured with the Sensirion SCD30 CO_2_ sensor, and data are being transferred wirelessly to a base station. The exhaled CO_2_ is measured in four directions at various distances from the head of the subject, and interpolated to create spatial heat maps of CO_2_ concentration. Statistical analysis using the Friedman’s analysis of variance (ANOVA) test is carried out to determine the validity of the null hypotheses (i.e., distribution of the CO_2_ is same) between different experiment conditions. Results suggest CO_2_ concentrations vary little with the type of mask used; however, improper use of the face mask results in statistically different CO_2_ spatial distribution of concentration. The use of low cost sensors with a visual interpolation tool could provide an effective method of demonstrating the importance of proper mask wearing to the public.

## 1. Introduction

The use of cloth face coverings and other types of face masks has become widely adopted in 2020–21 to reduce the emission of pathogens in exhaled breath, and mitigate the spread of infectious diseases such as influenza and more recently COVID-19. Wearing face coverings in public settings has also been the centre of political debate and discussions about human freedom. Since the beginning of the 2020 pandemic, there has been discussion on the efficacy of face coverings and masks. However, recent studies show clear benefits in reducing transmission [1,2,3,4], and the use of face coverings has been accepted by the wider scientific community. In one study, [5], two hairdressers both tested positive for COVID-19 and wore a double layer face mask while working. They did not pass the disease to their clients, while their family members did contract it. In [6], the authors compared 200 countries and concluded that in the countries where face masks were not recommended, the per-capita mortality increased each week by a factor of 1.619, or 61.9%. On the other hand, in countries recommending masks, the per-capita mortality tended to increase each week by just 16.2%. This, however, was based on the assumption that wearing face masks properly was adhered to or enforced. Another study [7] examined the infection rate in 15 states plus Washington D.C. after the government mandated the use of face masks in public and concluded that the COVID-19 growth rate declined by a maximum of 2 percentage points per day in these states. Evidence is also provided in a laboratory study [8], where animal experiments were conducted to show a barrier made from typical mask material reduced transmission of the SARS-CoV-2 virus.

Face coverings and face masks are predominantly a source control. During normal breathing, speech, coughing or sneezing, respiratory particles are exhaled over a wide range of sizes from under 1 µm to over 100 µm in diameter. Some of the larger droplets are visible and deposit rapidly due to gravity, while the much finer aerosols are carried in the exhaled breath and room ventilation, and remain in the air for long periods of time. Respiratory viruses such as SARS-CoV-2 can be transmitted through deposition of larger droplets onto mucous membranes, or when aerosols are breathed in by another person. In a recent work, the plume of exhaled air is visualised using imaging techniques [9], demonstrating the influence of a face covering on the direction and extent of the plume.

In the present work, CO_2_ sensors are used to create a quantitative spatial distribution of exhaled breath concentrations. CO_2_ is metabolically produced by humans during respiration, and hence can be used as a proxy for the exhaled breath plume. The concentration levels of CO_2_ in indoor environments depend on the building occupancy and ventilation system. With poor ventilation, high CO_2_ concentration can remain in the air [10] with a steady increase in concentration over time. We explore the use of CO_2_ sensor measurements as a simple approach to characterise the distribution of exhaled breath and the influence of a face mask. While CO_2_ is not representative of risk associated with large respiratory particles, it is a good proxy for the smallest aerosol particles, which could potentially lead to airborne virus transportation. CO_2_ concentration has been used as a proxy for airborne infection transmission modelling in a number of studies [11,12]. Moreover, the effects of high level CO_2_ on human health and cognition are reported in [13,14,15].

In this paper, four NonDispersive InfraRed (NDIR) based CO_2_ sensors are used in four directions and at different distances from a person’s face to measure CO_2_ during normal speech under different conditions. One condition of particular interest is the improper use of the face mask, which is when the mask covers the mouth, but the nose remains uncovered. Although the mouth is covered, it is of interest to demonstrate the reduction of face mask efficacy as a source control with improper use. The parameters considered through the tests are:

- CO_2_ concentration with proper and improper wearing of face masks.
- CO_2_ concentration at different distances from the face.
- CO_2_ concentration in different directions of the face.
- CO_2_ concentration in an unventilated and naturally ventilated room.

We present a methodology for using data from CO_2_ sensors to create an interpolated concentration map and obtain a smooth graphical representation. We use statistical approaches to determine whether spatial variation in concentrations within and between test cases is significant.

The rest of the paper is organized as follows: Section 2 presents the experimental methodology including the hardware and test conditions. Section 3 presents the interpolation approach and graphical representations of the data followed by the statistical analysis. Section 4 presents the discussion and conclusions.

## 2. Methodology

### 2.1. Hardware

In this study, we use our in-house custom made hardware solution to perform the measurements. This system is preferred over the off the shelf units such as [16,17], as synchronized data from multiple sensors were required for the analysis. The hardware consists of three main modules and a rechargeable battery combined to create a custom sensor unit on a printed circuit board (PCB). The sensor units were coupled together wirelessly to a laptop that provided us with synchronized live multi-point data, which were stored for analysis. The modules are described in the following subsections:

#### 2.1.1. Sensing

For CO_2_ sensing, the Sensirion SCD30 [18] was used, its NDIR detection enables highly accurate carbon dioxide measurement. The CO_2_ measurement range is between 0 and 40,000 parts per million (ppm), with an accuracy of ±(30 ppm + 3%). The sensor is capable of taking one measurement every two seconds while the response time is 20 s.

#### 2.1.2. Communication

XBee modules, based on the IEEE 802.15.4/Zigbee protocol [19] were used for wireless communication. This operates on the 2.4 Giga Hertz (GHz) industrial scientific medical (ISM) band with a range of 40 m indoors and are programmed to work in the Application Programming Interface (API) mode. The data from the sensor were collected by the microcontroller and transmitted to a coordinator that was connected to a laptop.

#### 2.1.3. Data Processing

**Figure 1:**
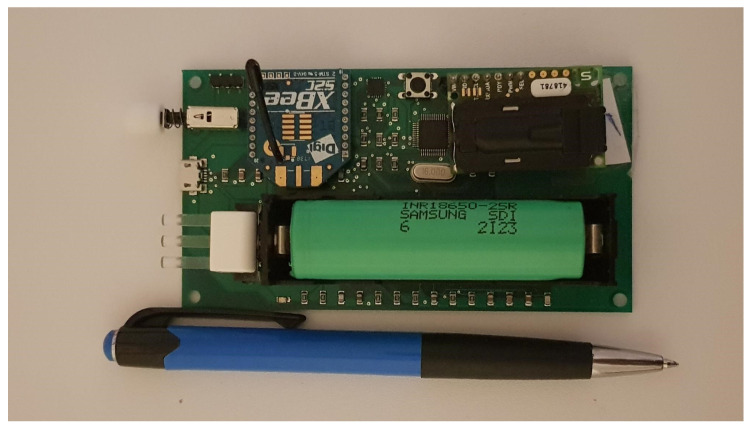
Sensor unit.

The 32-bit ARM cortex M3 STM32F103x microcontroller was used to collect and process data from the sensor, settings for the sensor, e.g., sensing frequency, pressure/temperature compensation, calibration factor were all coded into the microcontroller before implementation, full details of the microcontroller specification are available in [20]. The developed unit is shown in Figure 1.

#### 2.1.4. Power

The sensor unit is equipped with a 3.7 volt ‘18650’ rechargeable battery and a battery management chip embedded on the PCB. We use a 2.5 Ampere hour (Ah) capacity battery, which enables the unit to operate for several days before a recharge is required.

#### 2.1.5. Calibration

The sensor unit were calibrated against high end units in a controlled environment in our in house chamber with controlled CO_2_ exposure. It was observed that deviation of the sensor readings was within ±(30 ppm). Furthermore, after every test, the sensors were allowed to return to baseline values before the next test. This occasionally required the ventilation of the indoor environment between measurements.

### 2.2. Test Conditions

**Figure 2:**
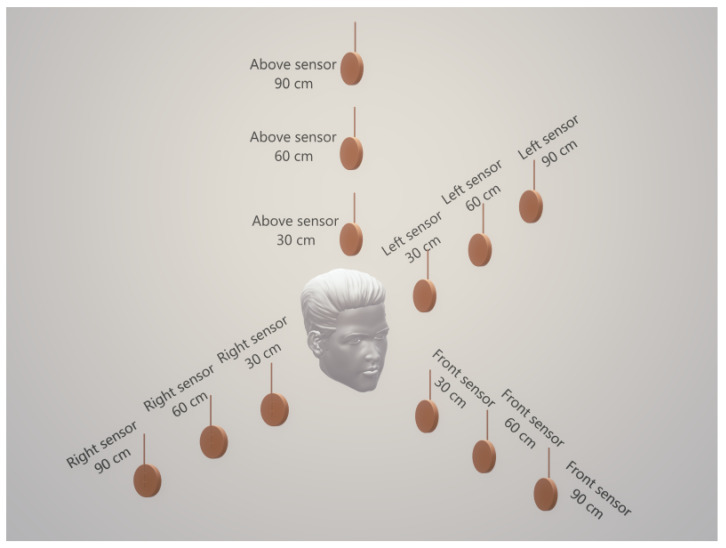
Experimental setup.

The tests were conducted in a residential 4 m × 3 m room with one window and two doors. The male subject was sitting in the middle of the room with sensors mounted on tripods in four directions; (i) in front of the face, (ii) on the right and left of the face, and (iii) a sensor hanging from the ceiling above the head. These tests were conducted with all sensors at 30, 60 cm and 90 cm distance from the face. The experimental setup is illustrated in Figure 2. For all sensor distances, tests were carried out with proper use of the face mask, i.e., the mouth and nose covered and improper use, i.e., with the mouth covered and nose uncovered. Four different types of masks were used in the experiments; these are (i) a single layer fabric face covering, (ii) a double layer fabric face covering, (iii) a surgical type mask, and (iv) a KN95 rated mask. Finally, all tests were performed in the unventilated room with the window and doors closed and with the window and one door open to provide ventilation. The sensors were programmed to take a measurement every two seconds for a period of 90 s. The subject was required to speak normally (counting from 1 to 90).

## 3. Results

This section presents the measured data, and the graphical representation of the spatially distributed data, followed by the statistical analysis of the measurements.

### 3.1. Summary of CO_2_ Measurements

**Figure 3:**
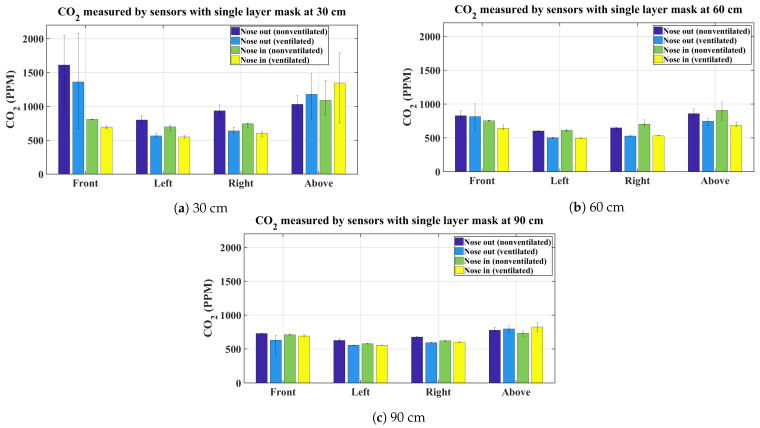
Average CO_2_ measurements using a single layer mask.

**Figure 4:**
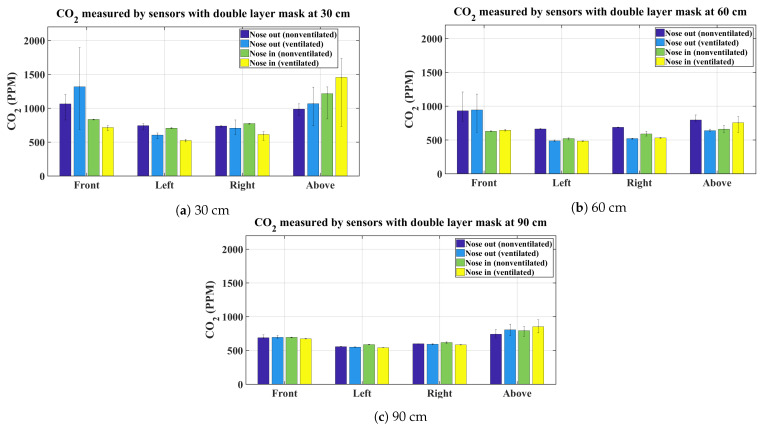
Average CO_2_ measurements using a double layer mask.

**Figure 5:**
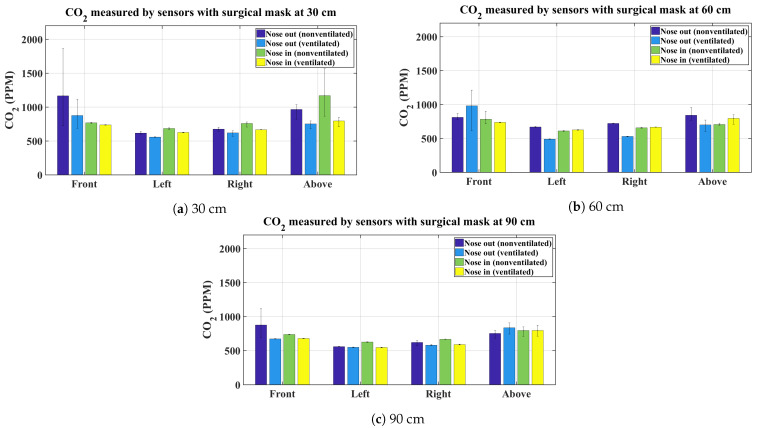
Average CO_2_ measurements using a surgical mask.

**Figure 6:**
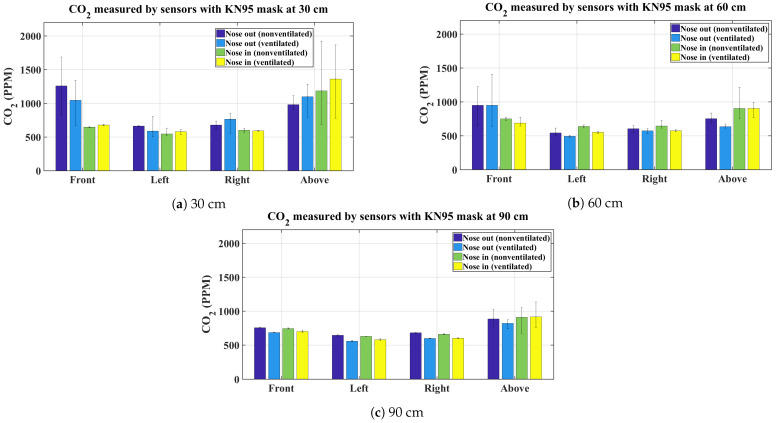
Average CO_2_ measurements using a KN95 mask.

**Figure 7:**
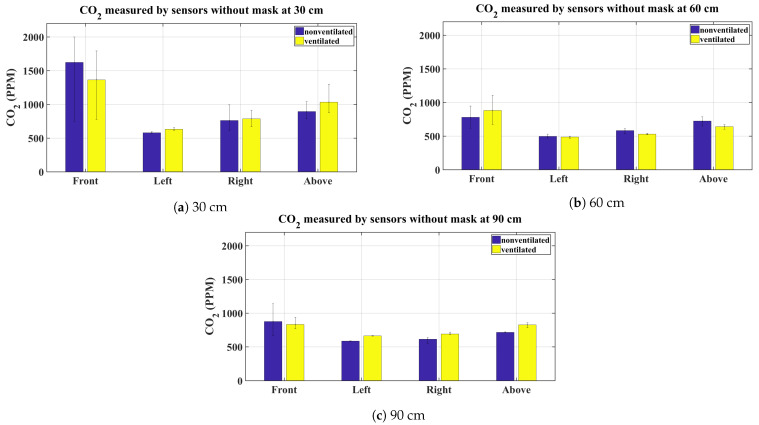
Average CO_2_ measurements without a mask.

The graphs shown in Figure 3, Figure 4, Figure 5 and Figure 6 present the average CO_2_ with error bars that indicate the maximum and minimum sensed gas concentration. The figures show the measurements for all mask types and all distances/directions. Average CO_2_ for both naturally ventilated and unventilated indoor environments are also displayed. In general, all masks follow a similar pattern. For reference, Figure 7 presents the sensor measurement when no mask is used.

Measurements obtained for the single layer fabric mask at 30 cm are given in Figure 3a. It is observed that speaking with the nose out registered notably higher CO_2_ concentrations at the sensor placed in the front of the face while lower values (close to baseline) are observed when the mask is properly worn. There are observable differences between the average readings obtained from the sensor at the front, in a ventilated and unventilated environment, for both the nose out and nose in case. The range of CO_2_ concentration at the sensor in front of the face is wide for the nose out case as shown by the error bars. The minimum values are low because the sensor is at baseline (700–800 ppm) just before the experiment starts (which is in fact the case for all experiments) and it reaches a maximum value during normal speech (2000–2200 ppm). The extremely high CO_2_ observed at a short distance is independent of the ventilation in the room. This is observed repeatedly with the different types of masks.

The left and right sensor show little to no increase in average CO_2_ concentration compared to baseline concentration. The error bars are also very close to the average values in all four cases for the left and right sensors. Interestingly, the sensor placed at the top of the head shows high CO_2_ concentration when the mask is properly worn with the nose in; this is due to the exhaled breath while being obstructed by the mask in the forward direction escapes in the cracks between the face and the mask and rises upwards due to it being warmer. As will be seen in the subsequent figures, this phenomenon is observed with every mask. At a distance of 60 cm the maximum CO_2_ concentration drops significantly by about ∼1000 ppm at the front sensors, this is due to the rapid dilution of the exhaled air plume. There is still some difference between the proper and improper use of the mask; however, the difference is not as pronounced as at 30 cm. Similar to the 30 cm case, the left and right sensors do not register any high CO_2_ values. The sensors above the head also show decreased CO_2_ concentration for all four cases. At 90 cm the obtained results are similar to the 60 cm case with only slight increase in the readings from the sensor above the head.

Figure 4a–c show the distribution of mean CO_2_ using a double layer mask. The results obtained are similar to the single layer mask. There is, however, marked difference at the front sensor in the nose out case for the ventilated and unventilated case. The unventilated case is showing lower mean values compared to the ventilated case. This phenomenon is not repeated by the other masks or this mask at other distances, and so potentially reflects experimental error. On the other hand, the sensor above the head again showed higher CO_2_ concentration, this is pronounced in the nose in (proper mask use) case, in which the exhaled air is blocked by the mask in the front and escapes to the top. Figure 4b,c show similar results to the 30 cm case.

Figure 5a–c show the test results for the surgical type mask. Results are similar to the single and double layer mask. Interestingly, evident from the narrow error bars, when these masks are properly worn, the measured CO_2_ displays negligible change from the baseline value. This is the same for all the mask types, an indication that all these masks have a comparable effect on the exhaled breath plume when worn correctly.

Figure 6a–c presents the results for the KN95 rated mask, here again the same pattern as for the other masks is followed and the results are consistent. For comparison, the average CO_2_ concentration when no mask is worn is shown in Figure 7. As expected, at 30 cm, high CO_2_ concentration is observed by the sensor located at the front and above the head. While there is little impact on the left and right sensors. Relatively lower CO_2_ values are observed at 60 cm and 90 cm.

To compare the best and worst case scenarios; the average CO_2_ measured with no mask on in the non-ventilated case at 30 cm at the front of the head is 1621 ppm, while this value for the properly worn KN95 mask under the same conditions is 645 ppm.

### 3.2. Spatially Interpolated CO_2_ Concentrations

In order to visualize the CO_2_ concentration distribution from a limited number of sensors, spatial interpolation between the sensor readings is used. In this study, the natural neighbour interpolation (NNI) [21] is used to interpolate and generate a smooth continuous surface from the distributed sensor readings. NNI is a local interpolation technique, which is based on the Voronoi tessellation of the data points or sensor readings. The Voronoi tessellation is a set of tiles (or polygons) where each tile surrounds one data point. Every point in a tile is closer to its corresponding data point than it is to any other data point. After the Voronoi tessellation is constructed, the NNI works on introduction of query points (non-measured points) which generate their own tiles, thus establishing a link between the non-measured and sensed points. The area of the new tile captures a segment of the old tiles and the interpolated value of the new tile is a weighted average of the neighbouring points, see [21] for detailed description. Let point ***x*** be the query point with *N* neighbours for which we wish to predict the CO_2_ concentration ${\hat{C}}_{x}$, then by denoting $A(x_{i})$ as the captured area from the neighbouring tile *i* and A(x) as the total area of the tile corresponding to query point x, the interpolated CO_2_ is given by

(1) $${\hat{C}}_{x}=\sum_{i=1}^{N}w_{i}C_{i}$$

where $C_{i}$ is the CO_2_ concentration of the neighbouring sensor *i* and its weights $w_{i}$ is given by

$$w_{i}=\frac{A(x_{i})}{A(x)}\;\;0\leq w_{i}\leq 1,\;\sum_{i=1}^{N}w_{i}=1.$$

**Figure 8:**
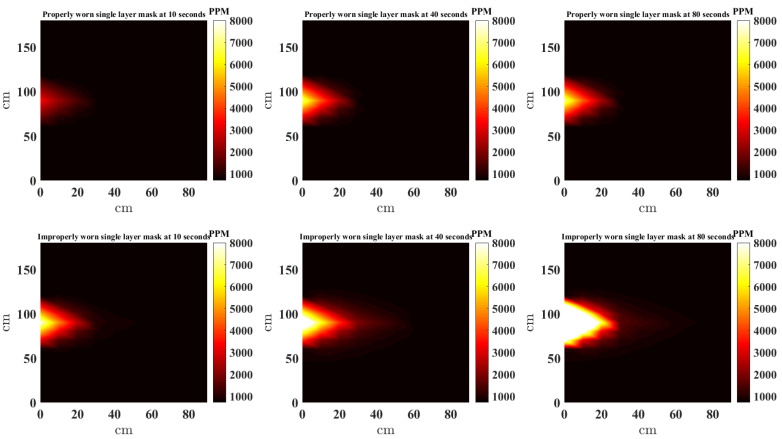
Interpolated CO_2_ with properly and improperly worn single layer mask.

**Figure 9:**
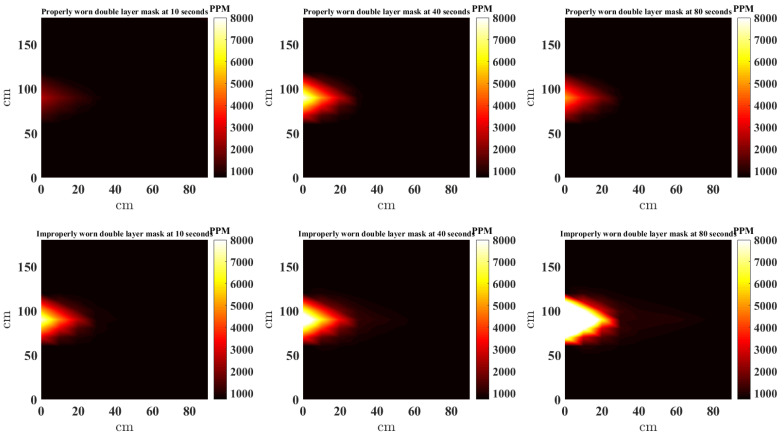
Interpolated CO_2_ with properly and improperly worn double layer mask.

**Figure 10:**
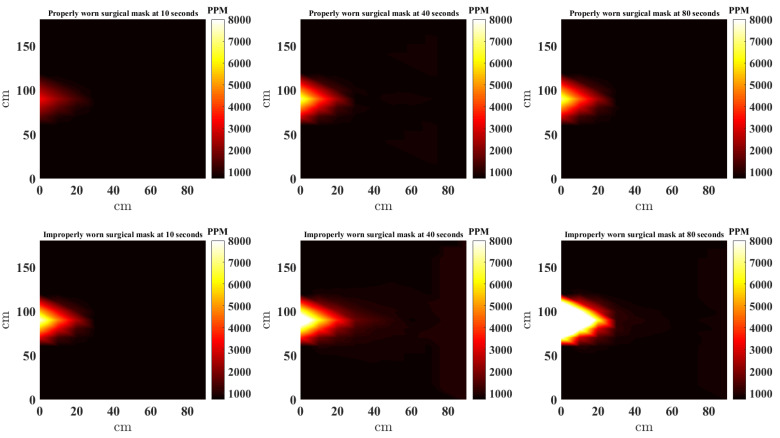
Interpolated CO_2_ with properly and improperly worn surgical mask.

**Figure 11:**
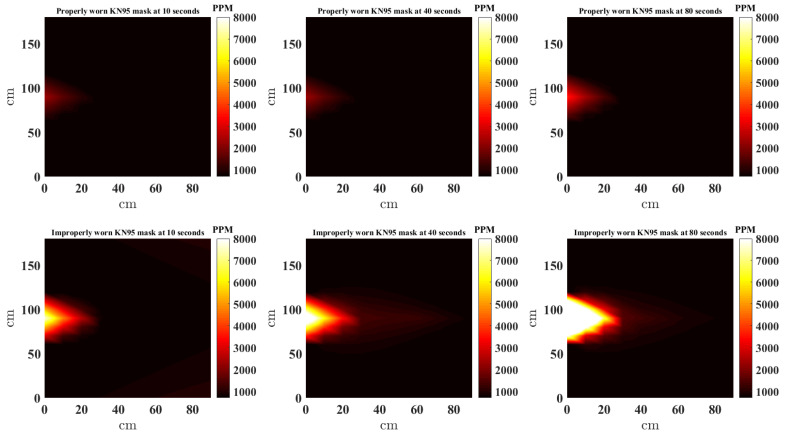
Interpolated CO_2_ with properly and improperly worn KN95 mask.

In this study, the scatteredInterpolant function [22] in MATLAB is used to to generate the NNI CO_2_ heat maps. Furthermore, to provide a complete picture, the estimates at locations outside the convex hull of the sensors are extrapolated using *linear extrapolation*. Figure 8, Figure 9, Figure 10 and Figure 11 present a top down view of the interpolated heat maps of the CO_2_ concentration. This 2D interpolation does not contain the measurement at the sensor above the head. The head is at location (0, 90) cm, while readings are taken from sensor located at (0, 0), (0, 30), (0, 60), (0, 120), (0, 150), (0, 180), (30, 90), (30, 60), (30, 90). It is noted that to avoid sensor error and offset between sensors, the same three sensors are used at the nine locations sequentially to obtain the results. The source CO_2_ is taken by placing the CO_2_ sensor in close proximity to the face (almost touching) and data recorded when the mask is worn properly and also when the nose is out. Figure 8, Figure 9, Figure 10 and Figure 11 show the heat map of CO_2_ distribution with proper and improper use of a single layer fabric, double layer fabric, surgical type and KN95 rated masks, respectively, in the unventilated setting. Snapshots at 10, 40 and 80 s are shown. When the mask is properly worn, the CO_2_ concentration is far less than when the mask is worn improperly. Moreover, the distance travelled by the plume is longer with improper mask use. It is interesting to note that when measuring for the source CO_2_ concentration close to the face with the mask properly worn, the sensor did register higher CO_2_, as can be seen in the first rows of Figure 8, Figure 9, Figure 10 and Figure 11. Whether this is a result of some CO_2_ escaping the mask material or through the cracks between the mask and face is unclear.

### 3.3. Statistical Analysis

In this section, the observed data are statistically analysed to determine if the means of the different experiment scenarios are statistically different. It is our understanding that the observed data do not follow a normal distribution; therefore, the non-parametric Friedman’s analysis of variance (ANOVA) test [23] is used.

#### Friedman’s ANOVA Test

The Friedman’s test is similar to the balanced two-way ANOVA test, but it is carried out on the ranks instead of the raw data. The approach tests values of ranks by columns (Treatment) while adjusting for the ranked rows (Blocks). The procedure checks for the null hypothesis in different treatment groups, which is valid if

$$H_{0}:\;{\bar{m}}_{1}=\ldots ={\bar{m}}_{k}$$

and is rejected when

$$H_{1}:\;{\bar{m}}_{1}\neq \ldots \neq {\bar{m}}_{k},$$

where ${\bar{m}}_{k}$ is the mean of the treatment group. For *k* treatment groups and *b* block groups, the Friedman’s statistic is given by

(2) $$F_{r}=\left[\frac{12}{b\left(k\right)\left(k+1\right)}\sum_{j=1}^{k}S_{j}^{2}\right]-3b\left(k+1\right)$$

where $S_{j}^{2}$ is the squared sum of ranks for sample treatment (column) *j* and $F_{r}$ follows as chi-square distribution. The null hypothesis is rejected when

(3) $$F_{r}>\chi_{a,k-1}^{2},$$

where the significance level a=0.05 is used in this paper. The *p*-value is obtained by

(4) $$P\left(\chi_{k-1}^{2}>F_{r}\right).$$

Table 1 shows the Friedman’s test results for two treatment scenarios, i.e., when the mask is properly worn and when it is improperly worn. In order to account for any mask bias, each mask forms a block resulting in four blocks. The test is carried out for naturally ventilated and unventilated environments. Furthermore, tests for all distances and directions are conducted for the null hypothesis.

For the unventilated environment, the null hypothesis for the front and above sensors are rejected for 30, 60, and 90 cm, signifying that the exhaled CO_2_ is statistically different between proper and improper mask use in the front and above the head regardless of the type of mask used. This is indeed expected and is evident from the bar charts in Figure 3, Figure 4, Figure 5 and Figure 6. On the other hand, for the same environment, the null hypothesis is not rejected for the left and right sensors at all distances, except for the left sensor at 30 cm. Again evident from the average bar charts presented earlier, significant difference in the average values is not observed on the left and right sides. Thus, exercising improper use of a mask does not have an impact on CO_2_ concentration on either side of the face. As pointed out, Friedman’s test on the left sensor at 30 cm did reject the null hypothesis however this is attributed to the negligible variation in the left (and also the right) sensor, which in fact does not register any increase in CO_2_ values above the baseline. This is evident from the narrow error bars over the left and right sensors in Figure 3, Figure 4, Figure 5 and Figure 6. This results in the statistical test misinterpreting the small inherent bias in the individual sensors as the basis for rejecting the null hypothesis. For the ventilated case, only the left sensor at 30 cm is not rejected while the null hypothesis for the rest of the cases is rejected. For the left and right sensors, statistical test results should not be accepted on face value and should be analysed in conjunction with Figure 3, Figure 4, Figure 5 and Figure 6, where it is seen that the left and right sensors register slightly different gas concentrations due to sensor bias and the same explanation as for the left and right sensors in the non-ventilated case applies for the ventilated case. The two smallest *p*-values are obtained when comparing proper and improper mask use at 30 cm in front of the face in both ventilated and non-ventilated environments. This signifies a substantial difference in observed CO_2_ measurements in these cases.

## 4. Discussion and Conclusions

In this paper, we have demonstrated the use of NDIR, CO_2_ sensors to quantitatively determine CO_2_ concentration distribution and highlight the importance of proper face mask use.

The Sensrion SCD30 sensor shows fairly accurate CO_2_ concentration, and we noticed fast response times. It was observed that when the human subject moved away after the experiment ended, the sensor took (15–20 s) to return back to baseline values. It is noted that although this study shows the concentration of exhaled breath relative to the mask fit on a persons face, it does not directly tell us about the virus concentration in the exhaled plume. The presented study has limitations in the sense that natural ventilation conditions are notoriously difficult to measure and control. Finally, due to the stringent lockdown rules in the UK in 2020, the study could not be extended on multiple subjects, and the experiments were performed on a single subject.

This study shows that CO_2_ measurement is a simple but effective way of looking at mask fit and such data could be used with sensor systems (such as the one we have developed) to guide mask fit in non-critical settings for the public. Indeed, similar results can be obtained with a thermal imaging cameras that have CO_2_ sensing built in; however, these are very expensive.

The key conclusions from this study are:

- There is a significant difference in the CO_2_ concentration when the face mask is used properly compared to when the nose is out during normal speech in the front and above the face.
- The type of mask used in this study had little to no affect on the CO_2_ concentration distribution.
- The CO_2_ concentration to the left and right of the face does not noticeably change from baseline values.
- CO_2_ rises above the face during normal speech, this is more pronounced when the mask is properly worn; however, CO_2_ at the front of the face is considerably reduced.
- At shorter distances to the speaker, the ventilation of the indoor environment does not impact CO_2_ concentrations.

The immediate practical implications of the study relate to the importance of proper mask wearing, demonstrating that a mask that does not cover the nose does not effectively contain the exhaled plume. While the study does not consider the impact on respiratory particles, it is reasonable to assume that wearing a mask in this way will compromise the effectiveness of source control both in terms of the viral particles that may be exhaled and the distance that they can travel in a highly concentrated plume. The findings also have potential implications for low cost approaches to demonstrate the importance of mask fit for public use. The use of spatially distributed CO_2_ sensors with a graphical interpolation interface may be an effective approach for training and education in the importance of correct mask wearing. Sensors similar to the one used in this study also have the potential to become vital tools in future studies such as smart ventilation, behavioural science and infection risk modelling.

## Figures and Tables

**Table 1:** Friedman’s test.

Treatment	Blocks	Environment	Direction	Distance	*p*-Value	Null Hypothesis
Nose in/out	All masks	Not ventilated	Front	30 cm	$3.5\times 10^{-33}$	Rejected
Nose in/out	All masks	Not ventilated	Above	30 cm	$4.3\times 10^{-13}$	Rejected
Nose in/out	All masks	Not ventilated	Left	30 cm	$1.2\times 10^{-10}$	Rejected
Nose in/out	All masks	Not ventilated	Right	30 cm	1	Not rejected
Nose in/out	All masks	Not ventilated	Front	60 cm	$1.7\times 10^{-22}$	Rejected
Nose in/out	All masks	Not ventilated	Above	60 cm	$8.2\times 10^{-05}$	Rejected
Nose in/out	All masks	Not ventilated	Left	60 cm	0.117	Not rejected
Nose in/out	All masks	Not ventilated	Right	60 cm	0.0547	Not rejected
Nose in/out	All masks	Not ventilated	Front	90 cm	$1.2\times 10^{-15}$	Rejected
Nose in/out	All masks	Not ventilated	Above	90 cm	0.0005	Rejected
Nose in/out	All masks	Not ventilated	Left	90 cm	1	Not rejected
Nose in/out	All masks	Not ventilated	Right	90 cm	1	Not Rejected
Nose in/out	All masks	Ventilated	Front	30 cm	$3\times 10^{-37}$	Rejected
Nose in/out	All masks	Ventilated	Above	30 cm	$1\times 10^{-05}$	Rejected
Nose in/out	All masks	Ventilated	Left	30 cm	0.0575	Not rejected
Nose in/out	All masks	Ventilated	Right	30 cm	$9.7\times 10^{-11}$	Rejected
Nose in/out	All masks	Ventilated	Front	60 cm	$8.3\times 10^{-21}$	Rejected
Nose in/out	All masks	Ventilated	Above	60 cm	$3.2\times 10^{-15}$	Rejected
Nose in/out	All masks	Ventilated	Left	60 cm	$5.6\times 10^{-6}$	Rejected
Nose in/out	All masks	Ventilated	Right	60 cm	$7.6\times 10^{-19}$	Rejected
Nose in/out	All masks	Ventilated	Front	90 cm	$1.9\times 10^{-12}$	Rejected
Nose in/out	All masks	Ventilated	Above	90 cm	$7.4\times 10^{-5}$	Rejected
Nose in/out	All masks	Ventilated	Left	90 cm	$1.7\times 10^{-10}$	Rejected
Nose in/out	All masks	Ventilated	Right	90 cm	$4.4\times 10^{-11}$	Rejected

## Data Availability

The underlying data is available at https://github.com/Nav-101/Multi_sensors_mask_study.git (accessed on 13 September 2021).

## Abbreviations

The following abbreviations are used in this manuscript:

NDIR	NonDispersive InfraRed
ANOVA	analysis of variance
PCB	printed circuit board
API	Application Programming Interface
NNI	natural neighbor interpolation
ISM	Industrial Scientific Medical
Ah	Ampere hour
CO_2_	Carbon dioxide
GHz	Giga Hertz
ppm	parts per million
cm	centimeter
